# Dual negative roles of C/EBPα in the expansion and pro-tumor functions of MDSCs

**DOI:** 10.1038/s41598-017-12968-2

**Published:** 2017-10-25

**Authors:** John R. Mackert, Peng Qu, Yongfen Min, Peter F. Johnson, Li Yang, P. Charles Lin

**Affiliations:** 1 Vanderbilt University Medical Center, Nashville, TN 37232 United States; 2 Center for Cancer Research, National Cancer Institutes, Frederick, MD 21702 United States

## Abstract

Myeloid-derived suppressor cells (MDSCs) are greatly expanded in cancer patients and tumor-bearing mice. They infiltrate into tumors and modulate the tumor microenvironment. In an effort to identify molecular mediators responsible for expansion and the tumor-promoting function of MDSCs, we discovered CCAAT/enhancer binding protein alpha (C/EBPα) expression was significantly reduced in MDSCs from tumor-bearing mice compared to non-tumor-bearing hosts. Tumor-conditioned medium down-regulated C/EBPα expression, suggesting tumor secreted factors inhibiting the gene expression. Consistent with the function of C/EBPα in regulating the balance between proliferation and growth arrest in hematopoietic progenitors, myeloid lineage specific deletion of C/EBPα resulted in significantly enhanced MDSC proliferation and expansion, as well as an increase of myeloid progenitors and a decrease of mature cells. In addition, deletion of C/EBPα in MDSCs enhanced the pro-angiogenic, immune suppressive and pro-tumorigenic behavior of these cells by upregulating the production of iNOS and arginase, as well as MMP-9 and VEGF. Accordingly, tumors growing in C/EBPα conditional null mice displayed greater MDSC infiltration, increased vascularization and accelerated tumor growth. Taken together, this study reveals dual negative roles of C/EBPα in the expansion as well as pro-angiogenic and immune suppressive functions in MDSCs.

## Introduction

The immune suppressive and tumor-promoting properties of MDSCs have been well established. MDSCs are immature myeloid cells, comprised of cells of the monocytic and granulocytic lineages at earlier stages of differentiation^1,2^. In mice, MDSCs are defined by the simultaneous expression of CD11b (Mac-1), a myeloid macrophage marker, and the granulocytic marker Gr-1. Furthermore, they lack or have reduced expression of markers of mature myeloid cells, low levels of major histocompatibility complex (MHC) class II and co-stimulatory molecules and suppress immune responses *in vitro* and *in vivo* ^1,2^.

MDSCs are increased in various pathological conditions including cancer^2^. MDSC accumulation has been documented in the peripheral blood of cancer patients with melanoma, head and neck, breast, colon, renal, and non-small cell lung cancers^3^. The accumulation of MDSCs is often associated with poor prognosis as increased circulating MDSC levels were shown to positively correlate with clinical cancer stage and metastatic tumor burden^4^.

MDSCs are markedly expanded in the peripheral blood, spleens and bone marrow of tumor-bearing mice, and their production and infiltration into tumors increased as tumor size increased and with the duration of tumor growth^5^. MDSCs are capable of suppressing tumor immunity through multiple direct and indirect mechanisms on T-cells, dendritic cells and natural killer cells^1,2^. They also directly promote tumor growth through mechanisms of tumor angiogenesis. MDSCs from tumor-bearing mice express higher levels of MMP9, thereby increasing the bioavailability of VEGF^5^ and have been reported to mediate tumor refractoriness to anti-VEGF treatment^6^. While researchers have long observed the dramatic expansion of MDSCs in tumor-bearing hosts, the molecular mechanisms regulating MDSC expansion under tumor conditions remain less clear.

CCAAT/enhancer binding protein (C/EBP) transcription factors belong to a six-member family of leucine-zipper DNA binding proteins involved in cell cycle regulation and differentiation in various cell types. C/EBPα is the founding member of the family and regulates the balance between cell proliferation and differentiation in hematopoietic and non-hematopoietic tissues^7^. When activated, C/EBPα induces differentiation through transcription of target genes^8^ and slows proliferation via its ability to inhibit mitotic growth^9^. Specifically, C/EBPα regulates hematopoiesis by inducing myeloid differentiation and inhibiting erythroid differentiation in primitive progenitors^10^. Loss of C/EBPα cell-cycle control activity increases myeloid progenitor proliferation^11^. C/EBPα has also been identified as a tumor suppressor in multiple tissues^12^ and mutations in the C/EBPα gene or disruption of C/EBPα function have been found in acute myeloid leukemia^13^. In this study, we demonstrate that C/EBPα is down regulated in MDSCs from tumor-bearing mice. Myeloid lineage specific deletion of C/EBPα resulted in increased MDSC production and pro-angiogenic gene expression in MDSCs. Thus, C/EBPα plays dual negative roles in MDSC expansion and MDSC-mediated tumor angiogenesis.

## Results

### Tumor conditions down-regulate C/EBPα expression in MDSCs

**Figure 1:**
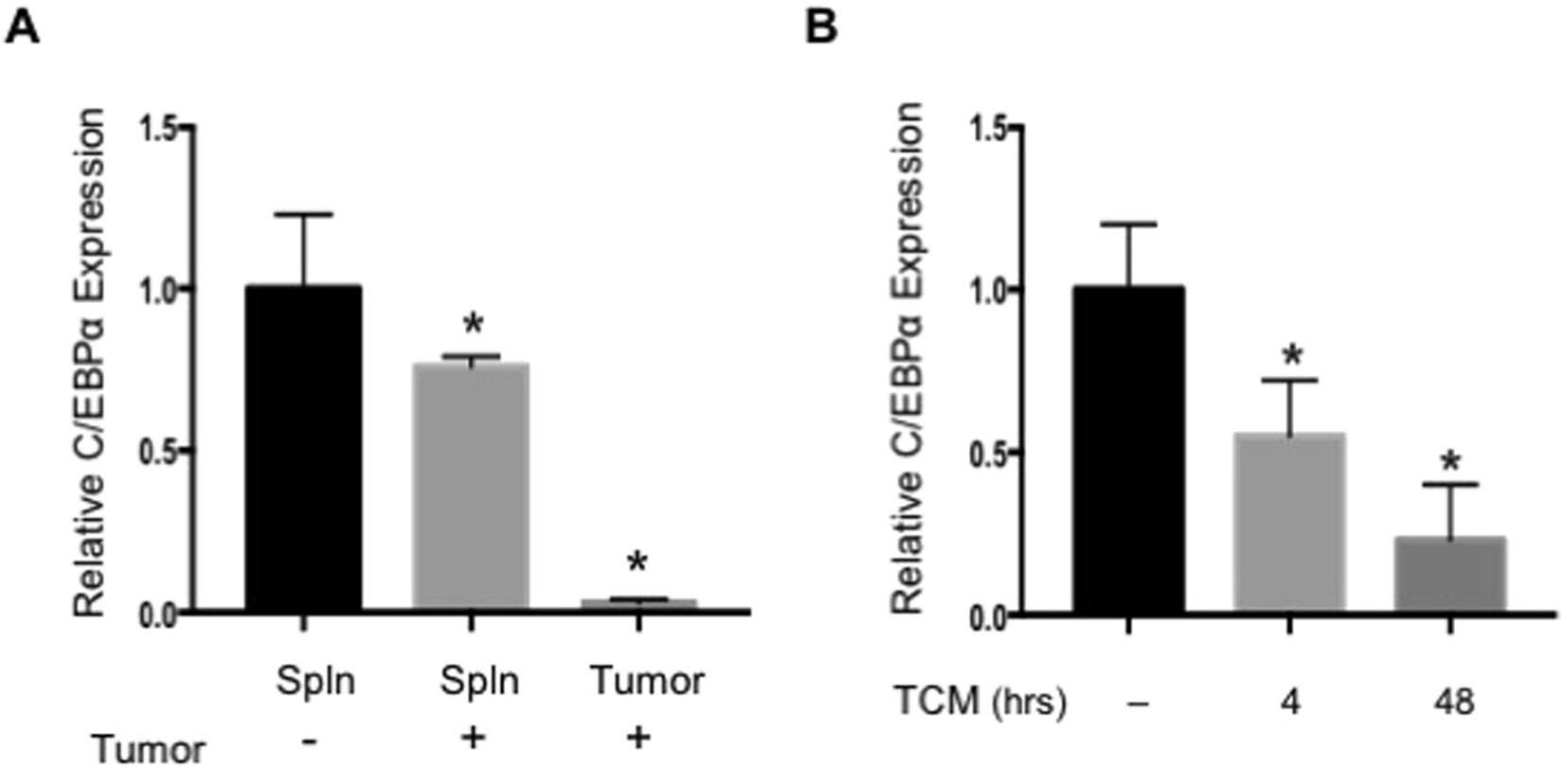
C/EBPα is down-regulated in MDSCs from tumor bearing mice. Gr-1+CD11b+ cells were isolated from spleens (spln) of C57BL/6 mice with (+) or without 3LL (−) tumors as well as tumor tissues (tumor). Gr-1+CD11b+ cells with greater than 95% purity were pooled from 5–7 mice, RNA was isolated and C/EBPα expression was measured by real-time PCR (**A**). Media was collected from 3LL tumor cells after 2–3 days in culture to make tumor-conditioned medium (TCM). 32D cells were cultured in a 50:50 mixture of TCM and fresh media for 4 or 48 hours. RNA was isolated and C/EBPα expression was measured by real-time PCR (**B**). *p < 0.05. The data are presented as mean with SD. The experiment was done in triplicate and repeated twice.

MDSCs are overproduced in cancer patients and tumor bearing animals. They infiltrate into tumors and promote tumor growth by promoting tumor angiogenesis and immune suppression. To better understand the regulation of MDSC expansion under tumor conditions, we compared gene expression in MDSCs isolated from spleens of C57BL/6 mice with or without a derivative of Lewis Lung Carcinoma (3LL) tumors. We found the expression of C/EBPα was significantly reduced in MDSCs isolated from the spleen of tumor-bearing mice compared to non-tumor bearing mice (Fig. 1A). Furthermore, C/EBPα expression was nearly undetectable in MDSCs infiltrated into tumor tissues (Fig. 1A). These findings demonstrated a negative regulation by tumor conditions on C/EBPα expression in MDSCs.

To confirm the *in vivo* finding, we examined the effects of tumor cell conditioned medium on C/EBPα expression *in vitro*. We cultured 32D murine myeloid cells in fresh medium or medium conditioned by 3LL tumor cells (TCM). C/EBPα expression was significantly reduced by more than 40% after 4 hours in TCM, and longer exposure (48 hr) to TCM reduced C/EBPα mRNA levels to 25% of normal (Fig. 1B). Similar results were observed in the human HL-60 myeloid cell line (data not shown). The finding suggests secreted factors from tumor cells are responsible for down regulation of C/EBPα in MDSCs.

### Genetic deletion of C/EBPα in myeloid lineage cells in mice accelerates tumor growth

Based on the critical role of C/EBPα in regulating the balance between proliferation and growth arrest in hematopoietic progenitors^7^, as well as down regulation of C/EBPα in tumor derived MDSCs (Fig. 1), we reasoned a negative function for C/EBPα in MDSC expansion and tumor promotion. To test the hypothesis *in vivo*, we utilized a system of conditional myeloid-lineage gene deletion. C/EBPα was deleted in cells of the myeloid lineage by breeding *Cebpa* ^flox/flox^ mice with *LysMCre* mice (CebpaΔ/Δ), which express Cre recombinase under the control of a murine lysozyme M promoter. Murine lysozyme M is exclusively expressed in cells of the monocyte/macrophage and granulocyte lineages of hematopoietic differentiation^14^ and its expression correlates with myeloid maturation, progressively increasing during myeloid differentiation^15^.

**Figure 2:**
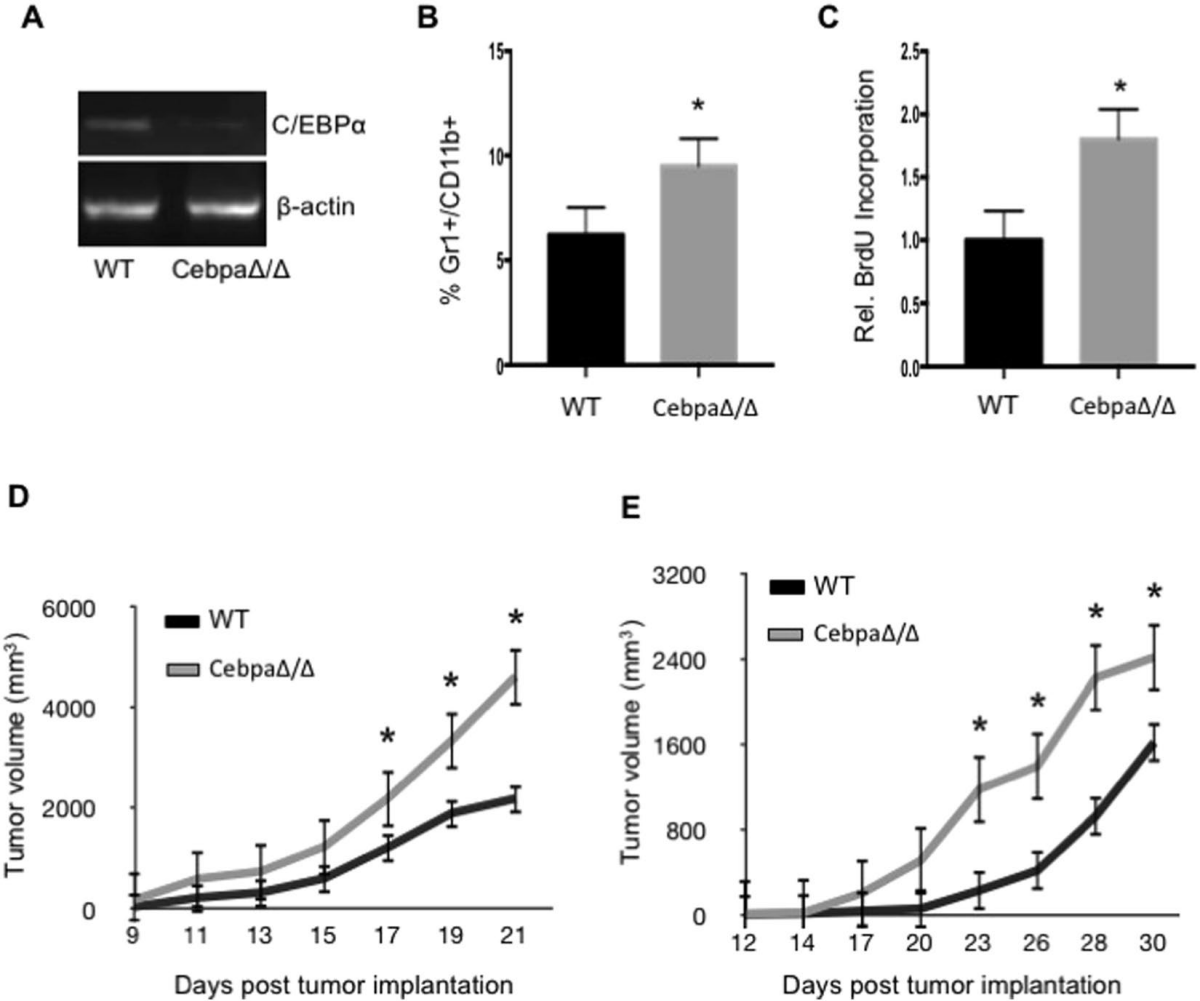
Tumor growth is accelerated in CebpaΔ/Δ mice. Gr-1+CD11b+ cells were purified from spleens of WT and CebpaΔ/Δ mice, RNA was isolated and C/EBPα expression was analyzed by semi-quantitative PCR (**A**). WT and C/EBPα CN mice were injected with 5 × 10^5^ 3LL (**B**–**D**) or B16 (**E**) tumor cells in the flank. After 21 days, spleens were isolated from the mice, processed into single-cell suspensions and stained with Gr-1 and CD11b fluorescent antibodies. The percentage of Gr-1+CD11b+ cells was analyzed by flow cytometry (**B**). 2 hours prior to sacrifice, the tumor bearing mice were injected with BrdU, and BrdU incorporation was measured in the Gr-1+CD11b+ cells by flow cytometry (**C**). Tumor dimensions were measured every 2–3 days with a caliper and tumor volume was calculated and plotted with time over time as indicated (**D** and **E**). *p < 0.05. n = 10 mice per group and repeated twice. The data are presented as mean with SD.

The conditional deleted mice are healthy, fertile and grossly normal. We isolated Gr1+CD11b+ MDSCs from the spleens of wild-type littermates and CebpaΔ/Δ mice and confirmed C/EBPα deletion by semi quantitative RT-PCR. In this system, C/EBPα was ablated in approximately 80% of the Gr1+CD11b+ MDSCs (Fig. 2A). This reduction in C/EBPα mRNA levels was similar to what was observed in MDSCs isolated from tumor tissues (Fig. 1A).

Next we examined the role of C/EBPα in MDSC expansion under tumor conditions; 5 × 10^5^ 3LL tumor cells were injected subcutaneously into the flanks of CebpaΔ/Δ and control litter mates for several weeks. Single cells suspensions from the spleens of mice with similar sized tumors were stained with anti-Gr-1 and anti-CD11b antibodies. Flow cytometry analysis revealed a significant increase in Gr1+CD11b+ MDSCs in tumor-bearing CebpaΔ/Δ mice compared to littermate controls (WT) (Fig. 2B).

Given that C/EBPα inhibits proliferation in myeloid progenitors^11^, we wondered if proliferation was greater in MDSCs from CebpaΔ/Δ mice. Prior to sacrificing the mice, mice bearing similar sizes of tumors were injected intra-peritoneally (i.p.) with BrdU. Flow cytometry analysis revealed that BrdU incorporation in MDSCs from CebpaΔ/Δ mice was nearly double that of littermate control MDSCs (Fig. 2C). These data suggest that C/EBPα inhibits MDSC expansion likely through inhibition of cell proliferation, and tumor conditions resulted in an expansion of MDSCs through down regulation of C/EBPα in myeloid cells.

To determine the function of myeloid C/EBPα in tumor growth, 5 × 10^5^ 3LL were injected subcutaneously into the flanks of CebpaΔ/Δ mice and control littermates. Tumor size was measured by a caliper. We found that the tumor growth rate was significantly increased in C/EBPα myeloid conditional null mice compared to littermate controls (Fig. 2D). Starting by Day 17, tumor volume was significantly greater in conditional null mice and this trend continued (Fig. 2D). We also inoculated CebpaΔ/Δ mice with 5 × 10^5^ B16 melanoma cells. An increased tumor growth rate was also observed with this model (Fig. 2E). Collectively, these findings indicate a negative role of C/EBPα in MDSC expansion under tumor conditions. Tumors down regulate C/EBPα in myeloid lineage cells that contribute to expansion of MDSCs and enhanced tumor growth.

### Tumors from C/EBPα myeloid conditional null mice have increased MDSC infiltration and angiogenesis

**Figure 3:**
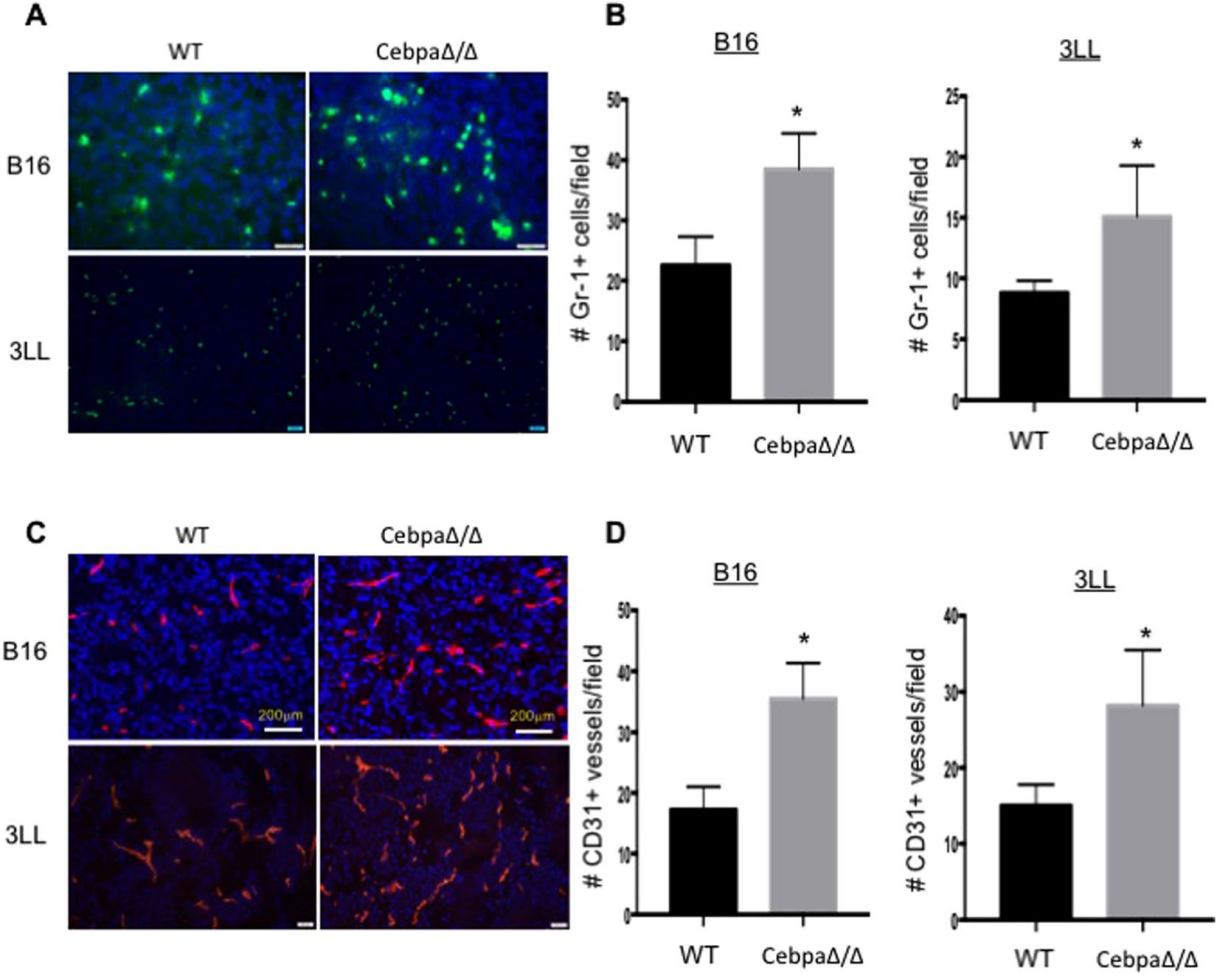
Tumors from CebpaΔ/Δ mice have increased MDSC infiltration and vascularity. Size matched B16 and 3LL tumors were harvested from CebpaΔ/Δ and control littermate mice, sectioned and stained with Gr-1 (**A**) or CD31 (**C**) antibodies. Representative images were shown. The number Gr-1+ cells (**B**) and CD31+ structures (**D**) were quantified in 10 randomly selected fields under microscopy. *p < 0.05. The data are presented as mean with SD.

MDSCs are known to infiltrate into tumors and modulate the tumor microenvironment to promote tumor progression^5^. As conditional deletion of C/EBPα in myeloid cells increased MDSC expansion and tumor growth, we then analyzed if the elevated MDSC expansion in spleens resulted in an increase of MDSCs in tumor tissues in mice with a myeloid-lineage deletion of C/EBPα. Tumors were harvested from CebpaΔ/Δ mice and control littermates 21 days after inoculation with 3LL or B16 tumor cells. Tumor sections were stained with anti-Gr-1 antibody (Fig. 3A) and Gr1+ cells were counted as a measure of MDSC infiltration. We observed a significant increase in MDSC infiltration into the tumors of C/EBPα conditional null mice relative to littermate controls in both tumor models (Fig. 3B).

It is well established that MDSCs directly promote tumor growth through increased tumor angiogenesis^5^. We therefore analyzed tumor vascular density by staining tumor sections harvested from both groups with an anti-CD31 antibody (Fig. 3C) and counted CD31+ blood vessels. A significantly higher blood vessel density was observed in the tumors grown in C/EBPα conditional null mice than controls in both tumor types (Fig. 3D). These data together support the hypothesis that tumor conditions down regulate C/EBPα expression in myeloid lineage cells resulting in an expansion and increased MDSC infiltration into tumors, where these cells promote tumor angiogenesis and tumor growth.

### C/EBPα negatively regulates the pro-angiogenic and pro-tumor activities of MDSCs

**Figure 4:**
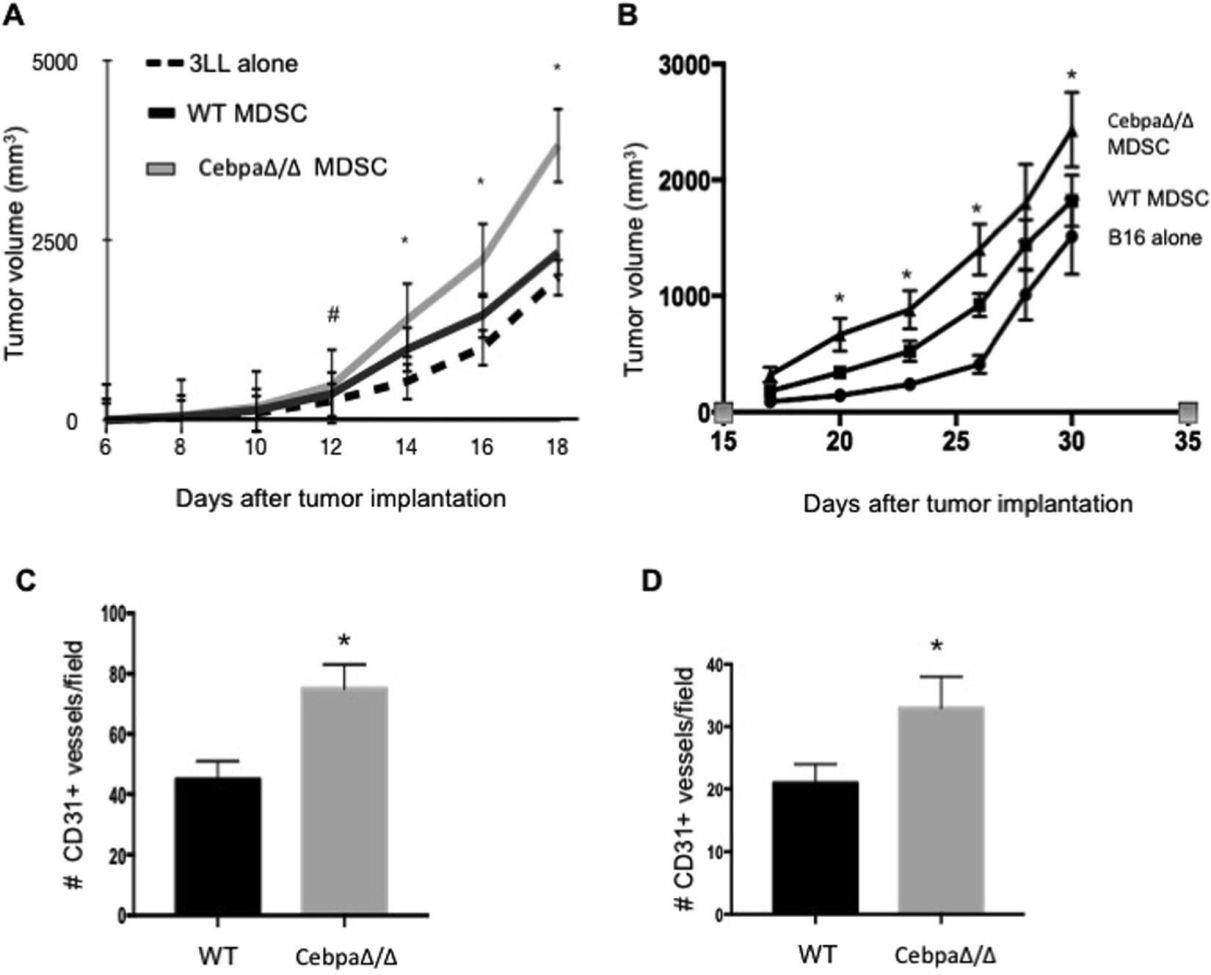
MDSCs from CebpaΔ/Δ mice exhibit enhanced activities in tumor angiogenesis and tumor growth. WT and CebpaΔ/Δ mice were injected with 1 × 10^5^ 3LL or B16 tumor cells in the flank. After 21 days, Gr-1+CD11b+ cells were purified from the spleens by FACS. WT C57BL/6 mice were injected with 5 × 10^5^ 3LL or B16 tumor cells alone, 3LL or B16 cells combined with 5 × 10^4^ Gr-1+CD11b+ cells from CebpaΔ/Δ mice or littermate WT controls. Tumor dimensions were measured every 2–3 days with a caliper and tumor volume was calculated and plotted with time (**A** and **B**). n = 10 mice per group and repeated once. Tumors were harvested, sectioned and stained with CD31 antibody. The number of CD31+ structures was quantified in randomly selected fields under microscopy of 3LL tumors (**C**) and B16 tumors. *p < 0.05. The data are presented as mean with SD.

To determine if MDSCs were responsible for increased tumor angiogenesis and accelerated tumor growth observed in CebpaΔ/Δ mice in which C/EBPα was deleted in myeloid lineage cells, we sorted Gr1+ CD11b+ cells from the spleens of CebpaΔ/Δ and control mice bearing 3LL and B16 tumors by FACS. We consistently achieved greater than 95% purity. Next, 5 × 10^4^ purified Gr1+CD11b+ cells were mixed with 5 × 10^5^ 3LL or B16 tumor cells (1:10 ratio) and co-injected subcutaneously into the flanks of C57BL/6 mice. C57BL/6 mice were also injected with tumor cells alone and tumor cells mixed with MDSCs isolated from WT mice as controls. Tumor size was measured by a caliper, and tumor volume was calculated. As expected, the tumor growth rate was greater when tumor cells were co-injected with Gr1+CD11b+ MDSCs from tumor-bearing WT mice compared to tumor cells alone (Fig. 4A and B). Interestingly, when mice were injected with tumor cells mixed with Gr1+CD11b+ MDSCs from tumor-bearing CebpaΔ/Δ mice, tumor growth was further accelerated (Fig. 4A and B). After an analysis of tumor vascularity by staining the tumor sections with CD31 antibody, we observed a significant increase in vascular density in the tumors co-injected with Gr1+CD11b+ MDSCs from CebpaΔ/Δ mice compared with controls (Fig. 4C and D). These data collectively support dual roles of C/EBPα in MDSC biology; it negatively regulates MDSC expansion, as well as the pro-angiogenic and pro-tumor activities of MDSCs. Deletion of C/EBPα in myeloid cells increased MDSC expansion, tumor infiltration and enhanced angiogenesis, and as a result, enhanced tumor growth.

### C/EBPα mediates the transition of myeloid progenitors to mature cells

**Figure 5:**
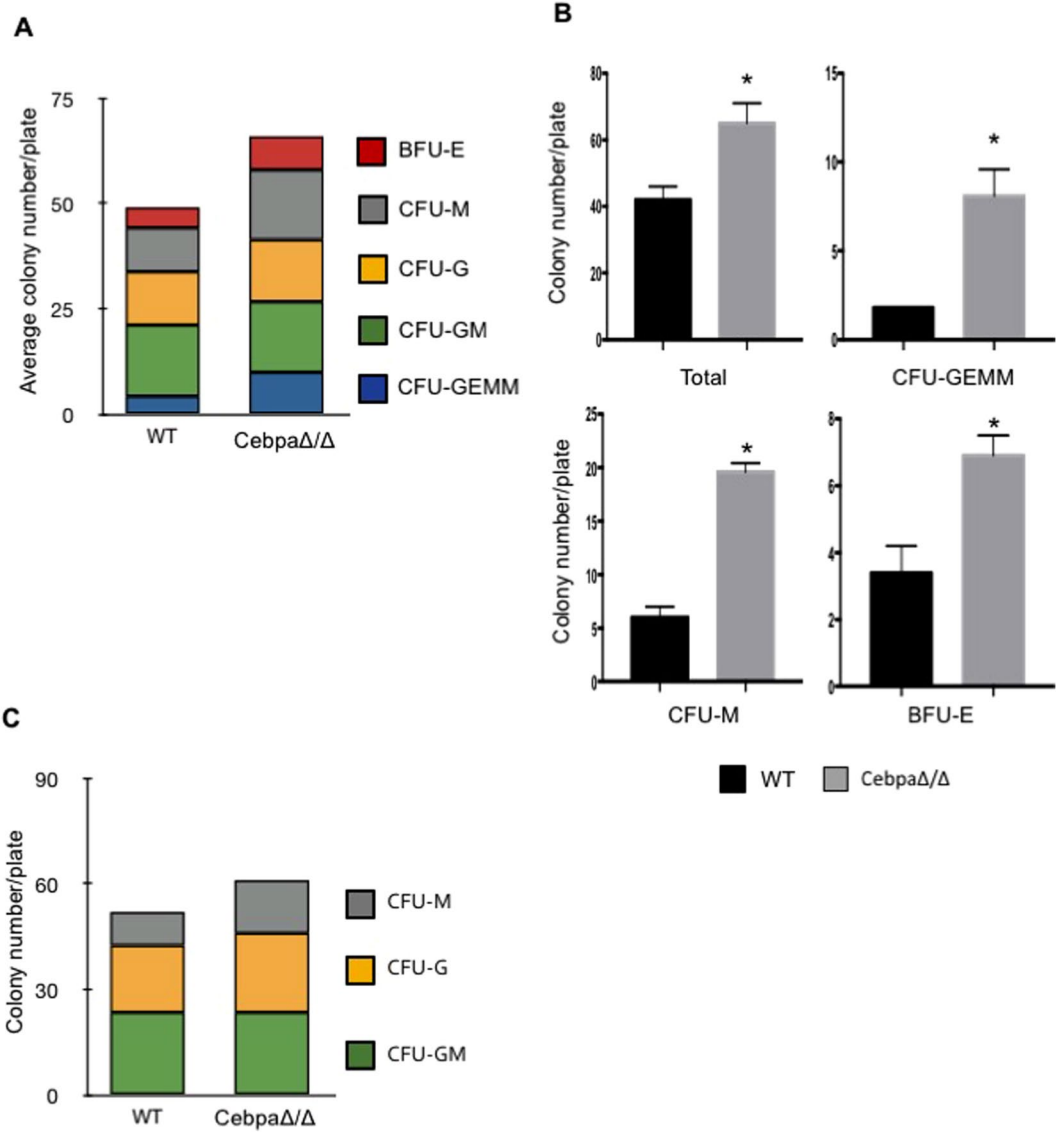
Myeloid progenitors are increased in C/EBPα conditional null mice. Bone marrow was isolated from CebpaΔ/Δ mice and littermate WT control mice. Single cell suspensions were made and cultured in MethoCult 3434 (**A**,**B**) or 3534 (**C**) semi-solid media. After 7–9 days incubation, colony types were evaluated and counted using an inverted microscope. The number of individual colony types and total colonies was quantified. Bone marrow was pooled from 3 animals each time and experiments were repeated twice. *p < 0.05. The data are presented as mean with SD.

Next, we attempted to determine the mechanism of C/EBPα in MDSC expansion. C/EBPα is known to inhibit proliferation and induce differentiation of myeloid progenitors^10^. Since MDSCs are immature myeloid cells, and deletion of C/EBPα led to an increase of MDSCs *in vivo*, it suggests a role of this transcription factor in myeloid cell maturation. To test the hypothesis, colony-forming unit (CFU) assays in semi-solid methylcellulose medium were performed. Single cell suspensions were prepared from bone marrow of CebpaΔ/Δ mice and littermate controls and cultured in methylcellulose for about a week. We observed a significant increase in the total number of colonies in bone marrow isolated from C/EBPα conditional null mice (Fig. 5A and B). The number of multi-potential progenitor (CFU-GEMM), macrophage (CFU-M) and erythroid (BFU-E) progenitor colonies was also significantly greater than littermate controls (Fig. 5B). Furthermore, in a similar CFU assay for GM (granulocyte-monocyte), M (monocyte) and G (granulocyte) progenitors, an increase in the total colony number and in the number and percentage of CFU-M progenitor colonies was observed (Fig. 5C).

We also analyzed the effect of myeloid-lineage C/EBPα ablation on circulating blood cells by performing complete blood counts (CBCs) on peripheral blood (Table 1). We found that the number of circulating monocytes was significantly decreased in CebpaΔ/Δ mice compared to controls. Other white blood cells were also decreased, although these differences did not reach statistical significance. Otherwise the CebpaΔ/Δ mice were not different from their littermate controls or from the normal range for their species, consistent with myeloid lineage specific deletion of C/EBPα. Taken together, these findings show that deletion of C/EBPα in myeloid cells yields an increase in myeloid progenitors and an accompanying decrease in circulating mature cells. The data suggest that down regulation of C/EBPα in myeloid cells blocks cell differentiation in addition to enhancing proliferation (Fig. 1), likely contributing to an expansion of MDSCs *in vivo*.

**Table 1:** Peripheral blood differential counts of myeloid conditional null mice and littermate WT mice.

	WT	CebpaΔ/Δ
RBC (M/mL)	10.15 ± 1.49	11.10 ± 0.45
Hemoglobin (g/dL)	13.13 ± 1.86	13.87 ± 0.50
HCT (%)	42.27 ± 5.48	45.23 ± 1.40
Total WBC (K/mL)	3.03 ± 1.78	2.89 ± 1.03
Lymphocyte (K/mL)	2.36 ± 0.23	1.66 ± 0.71
Neutrophil (K/mL)	1.23 ± 0.23	0.85 ± 0.26
Monocyte (K/mL)	0.45 ± 0.05	0.29 ± 0.09
Eosinophils (K/mL)	0.09 ± 0.05	0.06 ± 0.04
Basophils (K/mL)	0.04 ± 0.02	0.03 ± 0.02

Red blood cell **(**RBC), Hemoglobin (Hgb), Hematocrit (HCT), total white blood cell (WBC) and individual WBCs in peripheral blood were determined with an automated cell counter (HemaVet 960) in WT and CebpaΔ/Δ mice. Shown are the numbers from 3 mice ± SD. The experiment was repeated twice.

### C/EBPα negatively regulates angiogenic and immune suppressive activities of MDSCs

**Figure 6:**
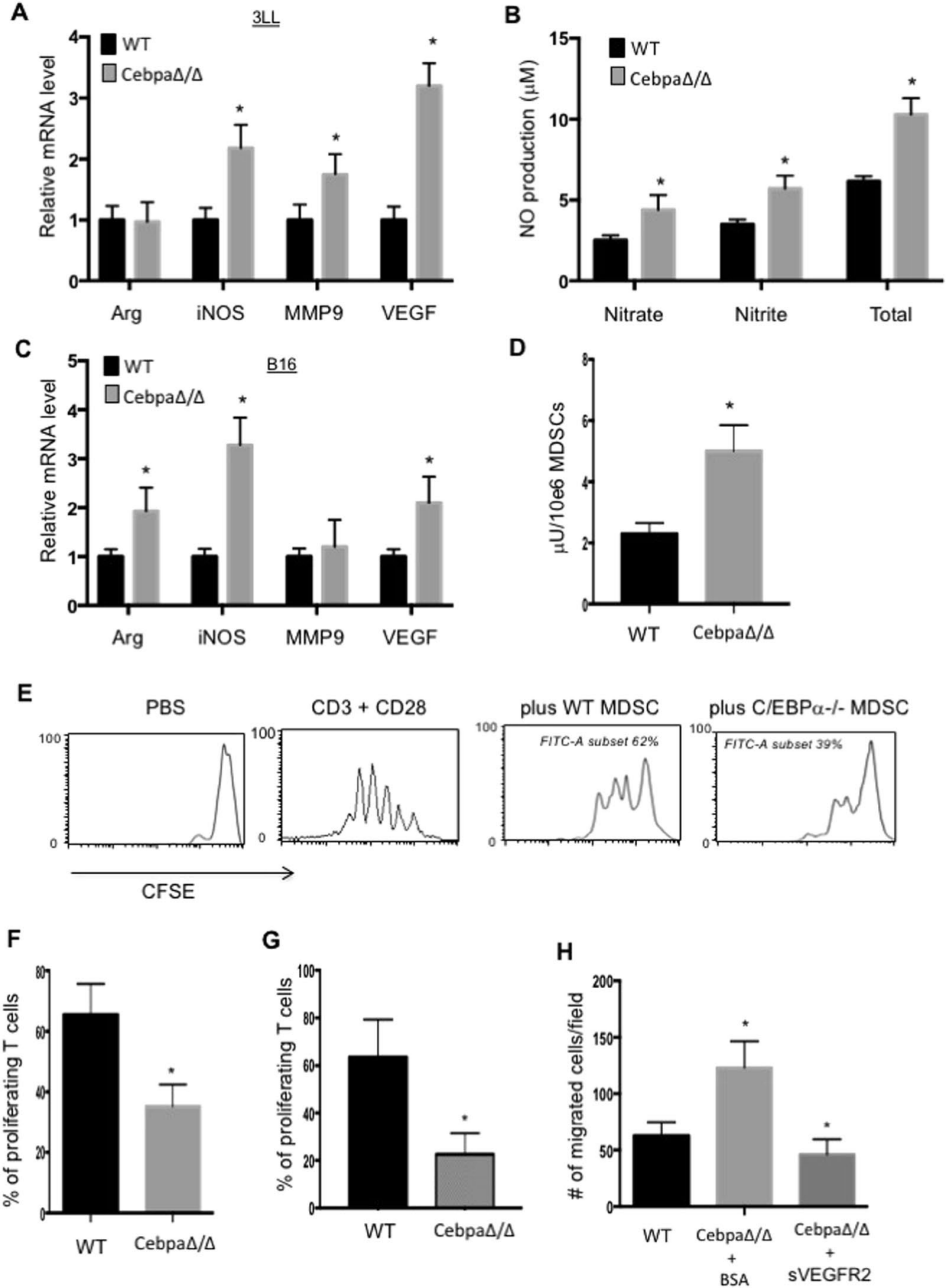
C/EBPα negatively regulates immune suppressive and angiogenic activities of MDSCs. Gr-1 +CD11b+ were magnetically purified from spleens of WT and CebpaΔ/Δ mice bearing 3LL tumor (n = 5 mice pooled together per group). RNA was isolated and Arg1, iNOS, MMP9 and VEGF expression was measured by qPCR (**A**). Gr-1+CD11b+ from 3LL tumor models were cultured for 3 days. Nitrate and nitrite production was measured in the culture supernatants with Nitrate/Nitrite Assay Kit and total NO production was calculated (**B**). Arg1, iNOS, MMP9 and VEGF expression was measured by qPCR in MDSCs isolated from B16 tumor models (**C**). Arginase activity of MDSCs purified from B16 tumor models were measured (**D**). *p < 0.05. The experiment was done in duplicate and repeated 3 times. CFSE-labeled normal CD4+T cells were stimulated with anti-CD3 mAb plus anti-CD28 mAb in the presence or absence of MDSCs isolated from WT or CebpaΔ/Δ mice for 3 days. PBS was used as a negative control. Proliferation of CD4+T cells was evaluated as CFSE dilution by flow cytometry and quantitated (**E** and **F**). A similar study as E and F was carried out using B16 tumor model (**G**). HUVEC cell migration in response to conditioned media collected from cultured MDSCs purified from WT and CebpaΔ/Δ mice (pooled from 3 mice per group) in the presence of control BSA or soluble VEGFR2 at 100 ng/ml were carried out using Transwell assays. Migrated endothelial cells were counted 6 hrs later (**H**) *p < 0.05. The experiment was done in duplicate and repeated 3 times.

MDSCs infiltrate into tumors and modulate the tumor microenvironment through production of growth factors and cytokines. Based on the findings that C/EBPα negatively regulates the tumor angiogenic and tumor-promoting activities of MDSCs (Fig. 4), we measured the expression of genes involved in MDSC-mediated immune suppression and angiogenesis. MDSCs were purified from the spleens of 3LL tumor-bearing mice by magnetic cell sorting. MDSCs shown to be greater than 95% pure (Gr1 and CD11b double positive) were further analyzed by qPCR. We found that C/EBPα ablation resulted in increased expression of inducible nitric oxide synthase (iNOS), but no effect on arginase 1 expression (Arg1) (Fig. 6A). iNOS activity, a mediator critical for immune suppressive functions of MDSCs, was also significantly increased in CebpaΔ/Δ MDSCs compared to controls (Fig. 6B). Additionally, we found that levels of matrix metalloproteinase 9 (MMP9) and vascular endothelial growth factor (VEGF), two important angiogenic mediators associated with MDSCs, were also significantly elevated in MDSCs from CebpaΔ/Δ mice (Fig. 6A). We also evaluated these findings using the B16 tumor model. We found that deletion of Cebpa in these MDSCs increased the expression of Arg1, iNOS and VEGF, but not MMP9 (Fig. 6C). Consistent with increased Arg1 levels, Arg1 enzymatic activity was increased as well in MDSCs isolated from B16 tumor bearing CebpaΔ/Δ mice compared to controls (Fig. 6D). These data provide molecular evidence supporting a negative role of C/EBPα in pro-angiogenic and pro-immune suppressive properties of MDSCs through inhibiting iNOS, Arg1, MMP9 and VEGF-A expression. The differences observed between these two tumor models regarding Arg1 and MMT9 expression in MDSCs illustrate that different model may utilize different mediators in regulating tumor angiogenesis and immune suppression.

Since iNOS and Arg1 are two potent immune suppression factors commonly associated with MDSCs, we further evaluated the immune suppressive activity of MDSCs. MDSCs purified from 3LL tumor bearing WT and CebpaΔ/Δ mice were co cultured with T cells, followed by analysis of T cell proliferation. Consistent with elevated iNOS, deletion of Cebpa in MDSCs increased anti T cell proliferation activity in these cells (Fig. 6E and F). Similarly, deletion of Cebpa in MDSCs isolated from B16 tumor bearing mice increased the immune suppressive activity of the cells compared to MDSCs isolated from WT mice (Fig. 6G). Additionally, we evaluated the angiogenic function of MDSCs using Transwell assays in combination of conditioned media collected from cultured MDSCs. We observed a significant increase in endothelial cell migration in response to the conditioned media collected from MDSCs isolated from CebpaΔ/Δ mice bearing 3LL tumors (Fig. 6H). Interestingly, blocking VEGF activity by using soluble VEGFR2 totally blocked the angiogenic function of MDSCs (Fig. 6H), which suggests VEGF plays a leading role in angiogenic activity of the MDSCs. A similar finding on angiogenic function of MDSCs was observed using the B16 tumor model (data not shown).

**Figure 7:**
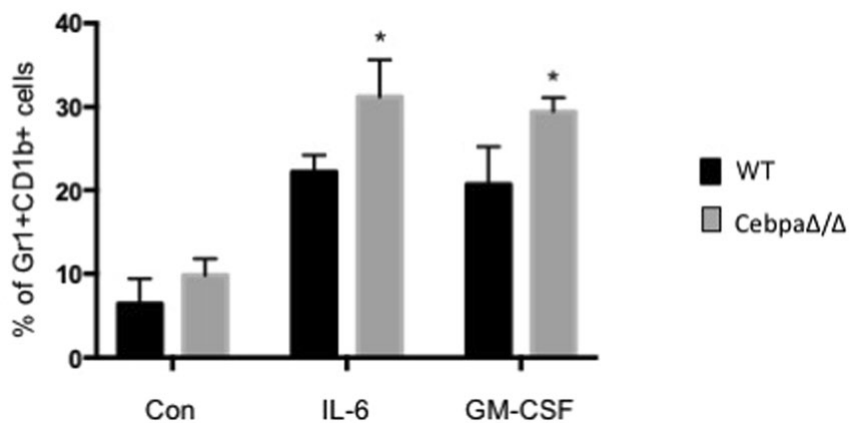
Deletion of C/EBPa in myeloid cells enhances the differentiation of monocyte to MDSC in response to IL-6 and GM-CSF. Monocytes purified from the blood of CebpaΔ/Δ mice and WT littermate were cultured in the presence of 0.2 ug/ml IL-6 or GM-CSF, respectively, for three days. CD11b+GR-1+ cells were quantitated by flow cytometry. Data are reported as mean ± SD from three independent experiments. *p < 0.05.

IL-6 and GM-CSF are two known factors regulating MDSC expansion associated with tumor conditions. We therefore attempted to evaluate the interaction of Cebpa with these two factors on MDSC differentiation. Purified monocytes from blood of WT and CebpaΔ/Δ mice were induced to differentiate to MDSCs in the presence of IL-6 or GM-CSF. We observed a significant increase of MDSC differentiation in Cebpa null cells compared to WT cells (Fig. 7). These data indicate Cebpa is one of the mediators responsible for MDSC expansion.

## Discussion

Advances in cancer treatment have made significant progress in recent years. However, tumors have proven frustratingly adept at evading therapeutics. MDSCs are highly elevated in peripheral blood of cancer patients, as well as tumor bearing animal models. They infiltrate into tumors and promote tumor angiogenesis through regulation of VEGF-A bioavailbility via MMP9^5^, and confer refractoriness to anti-VEGF treatment^6^. These cells have long been known to suppress the host immune response to cancer. As such, MDSCs make an attractive therapeutic target in the treatment of solid tumors. Here we present evidence that C/EBPα plays a negative role in MDSC expansion and the pro-tumor activities of these cells. C/EBPα expression is down-regulated in MDSCs from tumor-bearing mice and in myeloid cells cultured under “tumor conditions”. Tumor growth was significantly accelerated in mice harboring a conditional deletion of C/EBPα in myeloid-lineage cells. Furthermore, MDSC production was enhanced in the spleens of these mice and the tumors had greater MDSC infiltration and increased vascular density.

MDSCs from tumor-bearing mice have been shown to directly promote tumor angiogenesis and growth^5^. When MDSCs from mice with myeloid-lineage ablation of C/EBPα were combined with tumor cells in a co-injection study, tumor angiogenesis and growth was further increased, suggesting a specific role of C/EBPα in MDSC biology. Genetic deletion of C/EBPα results in an increase in immature myeloid cells and a complete lack of mature granulocytes. Conditional deletion of C/EBPα in our system yielded similar results. We saw an increase in the number and percentage of myeloid progenitors in the bone marrow and a decrease in the number of circulating monocytes in the peripheral blood of these mice. C/EBPα regulates cell-cycle progression through many mechanisms including E2F inhibition^16^. We observed nearly a two-fold enhancement in the proliferative capacity of MDSCs isolated from tumor-bearing mice lacking C/EBPα in myeloid lineage cells. This may partially explain why they were able to promote tumor growth to a greater extent than MDSCs from wild-type tumor-bearing mice.

The pro tumor properties of MDSCs are two folds, immune suppression and pro tumor angiogenesis. MDSC as its name reflects is well known for its immune suppressive functions, which are largely due to elevated arginase and iNOS in MDSCs associated with tumors. Our findings provide a molecular explanation of increased expression of the two factors in the tumor conditions, which suppress the expression of C/EBPα in MDSCs. Deletion of C/EBPα in these cells increases the levels of both factors in two tumor models tested in the study. Consistently, C/EBPα null MDSCs exhibit increased immune suppressive activities that likely contribute to increased tumor growth in the C/EBPα myeloid knockout mice.

It is well documented that MDSCs infiltrate into the tumor site and promote tumor angiogenesis through increased production of MMP-9 and VEGF^5,17^. Similarly, our findings provide a molecular explanation of increased expression of the two factors in the tumor environment. Tumor conditions down regulate C/EBPα in MDSCs, which leads to elevated expression of VEGF and MMP9 as well as increased angiogenesis. Together, our findings reveal negative roles of this transcription factor in the proangiogenic and immune suppressive properties of MDSCs. Accordingly, deletion of C/EBPα in myeloid cells results in a pro tumor progression phenotype.

The ability of MDSCs to promote tumor growth and metastasis has made uncovering the regulators of MDSC expansion and function a priority. While we have a good understanding of the molecules crucial for MDSC biology, in particular STAT3, much less is known about the negative regulation of MDSCs. A study determined that a micro-RNA, miR-223, suppresses accumulation of tumor-induced CD11b+Gr1 MDSCs^18^. This coincides with the data presented here, as miR-223 is a known C/EBPα target gene; C/EBPα up-regulates miR-223 expression during granulocyte differentiation^19^. Another article identified interferon (IFN) regulatory factor 8 (IRF-8) as a potential inhibitor of MDSC production and demonstrated that modulation of IRF-8 levels in tumor-induced CD11b+Gr-1+ cells can significantly abrogate their pro-tumorigenic behavior^20^. IRF-8 is a hematopoietic transcription factor that facilitates myeloid differentiation^21^. Interestingly, forced IRF-8 expression in a model of CML, BCR/ABL-transformed myeloid cells, specifically restored C/EBPα expression^22^. To our knowledge, this is the only report linking IRF-8 and C/EBPα expression and more work is needed to determine the specific role of these transcription factors in inhibiting MDSC expansion and activation. Furthermore, a study indicated a role for C/EBPβ in the immune suppressive functions of MDSCs. Marigo *et al*. provided evidence that hematopoietic deletion of C/EBPβ, a member of the same family of proteins as C/EBPα, led to a decrease in the number of MDSCs and a loss in the tolerance promoting activities of MDSCs, accompanied by a difference in differentiation of myeloid cells^23^. Very recently, we reported that C/EBP-δ, another C/EBP family member, plays important roles in MDSC expansion and tumor angiogenesis^24^. These findings, along with our current findings, point to important roles for this family of transcription factors in MDSC biology.

Therapeutic targeting of MDSCs in cancer and other pathological conditions is an area of intense and ongoing research. Recently proposed strategies include promoting myeloid-cell differentiation, inhibiting MDSC expansion, inhibiting MDSC function and elimination of MDSCs^2^. Our work suggests that restoring C/EBPα expression in MDSCs may promote their differentiation while inhibiting MDSC expansion and function. The validity of this approach has already been demonstrated in the myeloid leukemia field, where reintroduction of C/EBPα in CD34 + leukemic cells impaired their self-renewal capacity and enhanced myeloid differentiation^25^. And while their strategy of re-expressing C/EBPα by viral transduction is not the most feasible therapeutic option in cancer patients, restoring C/EBPα expression by another means may be a viable therapy and warrants further study.

## Materials and Methods

### Mice and cell lines

All mouse studies have been conducted according to Animal Welfare Act and the Public Health Service Policy and approved by Vanderbilt University Institution Animal Care and Use Committee (IACUC) and NCI. The animals were housed in pathogen-free units, in compliance with IACUC regulations. C57BL/6 J and LysMCre mice were purchased from Jackson Labs. Mice with a floxed *Cebpa* gene, called *Cebpa* ^flox/flox^, were developed as described^26^. We generated mice with myeloid-specific deletion of *Cebpa* by breeding *Cebpa* ^flox/flox^ mice to *LysMCre* mice, which express Cre recombinase under the control of murine lysozyme M promoter. C/EBPα^flox/flox^;LysMCre (+/−) (CebpaΔ/Δ) and littermate control mice C/EBPα^flox/flox^; LysMCre (−/−) (WT) were used in the study.

The 32D myeloid cell line, Lewis lung cancer derivative cell line (3LL), B16 melanoma cell line and HUVECs were purchased from ATCC and maintained per standard cell culture techniques.

B16 or 3LL cells (5 × 10^5^ cells), with or without purified Gr1+CD11b+ cells (0.5 × 10^5^ cells), were injected subcutaneously (s.c.) into the left flank of C57Bl/6 mice. The size of tumors was determined by measurement of tumor dimensions at 2–3 day intervals using a caliper. The equation volume = length × (width)^2^ × 0.5 was used to calculate tumor volume.

### MDSC isolation and flow cytometry

Tumor and spleen tissues were prepared into single cell suspensions. Single cell suspensions were stained with florescence-conjugated Gr-1 and CD11b antibodies (BD Biosciences), then sorted with a BD FACSAria cell sorter or analyzed using a BD LSRFortessa or BD FACScan in the VA or VUMC Flow Cytometry Resource as described^5,17^.

### Methylcellulose colony assays

Bone marrow from 2–3 mice was pooled and plated in methylcellulose semi-solid medium (Stemcell Technologies) supplemented with IL-3, IL-6, and stem cell factor with and without erythropoietin. After 7–8 days incubation at 37 °C, colonies were scored under an inverted microscope.

### *In vitro* analysis of MDSC activities

RNA was isolated from cells using the RNeasy Kit from Qiagen per the manufacturer’s instructions. cDNA was synthesized with the iScript cDNA Synthesis Kit (BioRad). For Real Time PCR, SsoFast EvaGreen Supermix (BioRad) and a CFX96 or MyiQ machine (BioRad) were used to analyzed the mRNA levels of Arg1, iNOS, MMP9 and VEGFA.

NO production was measured in culture media using a Nitrate/Nitrite Assay Kit (Alexsis Biochemicals) according to manufacturer’s instructions.

For arginase activity, purified MDSCs were lysed for 30 min at room temperature with 50 µL of Triton X-100 /PBS containing 5 µg pepstatin, 5 µg aprotinin and 5 µg antipain protease inhibitors. Subsequently, 50 µl of 10 mM MnCl_2_ and 50 mM Tris-HCl were added, and the enzyme was activated by heating for 10 minutes at 56 °C. Arginine hydrolysis was conducted by incubating the lysate with 100 µL of 0.5M L-arginine at 37 °C for 1 h. The reaction was stopped with 400 µL of H_2_SO_4_ /H_3_PO_4_/H_2_O (1:3:7, v/v/v). The concentration was measured at 540 nm. One unit of enzyme activity is defined as the amount of enzyme that catalyzes the formation of 1 µmol urea per minute.

For immune suppression assay, CD4+ T cells isolated from the WT spleens with CD4 mAb-coated magnetic beads were labeled with carboxyfluorescein diacetate succinimidyl diester (CFSE) (Molecular Probes), followed by stimulation with anti-CD3 mAb plus anti-CD28 mAb for 3 days in the absence or presence of MDSCs isolated from the spleens of WT or C/EBPα conditional KO mice bearing 3LL or B16 tumors. The ratio of MDSC:CD4+ T cells were 1:5. CD4+ T cell proliferation was evaluated by flow cytometry.

For endothelial cell migration assay, MDSCs were purified from the spleen of 3LL or B16 tumor bearing WT and CebpaΔ/Δ mice, and cultured in serum free RPMI overnight to collect conditioned media. Endothelial cell migration was assayed by Transwells as described^27^ with seeding HUVECs in the top chamber and condition media in the presence of BSA or soluble VEGFR2 in the bottom chamber.

### BrdU labeling

BrdU labeling was performed with the BD Pharmingen FITC BrdU Flow Kit (BD Biosciences). Mice were injected intraperitoneally with 2 mg of BrdU. After 2 hours, BrdU incorporation in Gr1+CD11b+ MDSCs was measured on a BD LSRFortessa cell analyzer.

### Immunohistochemistry

Frozen tumor sections were analyzed by immunohistochemistry following standard procedures^5,17^.

### Induction of Gr1+CD11b+ cells from monocyte

Monocytes were purified from blood of C/EBPα conditional null and WT littermate using EasySep™ Mouse Monocyte Isolation Kit (Stemcell Tech, Cal# 19861). Purified monocytes were cultured in RPMI-1640 in the presence of 0.2 ug/ml of IL-6 or GM-CSF, respectively, for three days. Vehicle treated cells were used as a control. CD11b+GR-1+ cells were quantitated by flow cytometry. Data are reported as mean ± SD from three independent experiments.

### Statistical analysis

All data were analyzed using the Student’s t test and were expressed as mean ± standard error.
